# Real-Time Spatial Mapping in Architectural Visualization: A Comparison among Mixed Reality Devices

**DOI:** 10.3390/s24144727

**Published:** 2024-07-21

**Authors:** Tam Le Phuc Do, Kang Sanhae, Leehwan Hwang, Seunghyun Lee

**Affiliations:** 1 Department of Immersive Content Convergence, Kwangwoon University, Seoul 01897, Republic of Korea; tamdlp@kw.ac.kr (T.L.P.D.); shluv7@kw.ac.kr (K.S.); optics.hwang@kw.ac.kr (L.H.); 2 Ingenium College, Kwangwoon University, Seoul 01897, Republic of Korea

**Keywords:** architectural visualization, augmented reality, HoloLens 2, iPhone, MR device, spatial mapping, photogrammetry

## Abstract

Recent advancements in communication technology have catalyzed the widespread adoption of realistic content, with augmented reality (AR) emerging as a pivotal tool for seamlessly integrating virtual elements into real-world environments. In construction, architecture, and urban design, the integration of mixed reality (MR) technology enables rapid interior spatial mapping, providing clients with immersive experiences to envision their desires. The rapid advancement of MR devices, or devices that integrate MR capabilities, offers users numerous opportunities for enhanced entertainment experiences. However, to support designers at a high level of expertise, it is crucial to ensure the accuracy and reliability of the data provided by these devices. This study explored the potential of utilizing spatial mapping within various methodologies for surveying architectural interiors. The objective was to identify optimized spatial mapping procedures and determine the most effective applications for their use. Experiments were conducted to evaluate the interior survey performance, using HoloLens 2, an iPhone 13 Pro for spatial mapping, and photogrammetry. The findings indicate that HoloLens 2 is most suited for the tasks examined in the scope of these experiments. Nonetheless, based on the acquired parameters, the author also proposes approaches to apply the other technologies in specific real-world scenarios.

## 1. Introduction

In an era dominated by advanced ICT technology, which serves as a cornerstone for myriad applications across diverse domains, emerging products aim to enhance productivity in conventional tasks. The evolution of data collection and transmission, particularly of spatial data, has undergone significant transformations. Various technologies have been developed to streamline data collection and reconstruct real-world environments, thereby augmenting the user experience and bridging the gap between virtual and physical realms [1,2,3,4]. With recent advancements in mixed reality (MR) technology, a surge in the utilization of realistic content has been noted. Augmented reality (AR), which seamlessly integrates virtual information with the real world, is recognized as an effective method for achieving realism. This technology overlays virtual objects onto actual surroundings, ensuring alignment with real-world coordinates [5,6]. Consequently, the successful implementation of AR depends on the real-time integration of virtual data with the physical environment. To achieve this, MR devices must accurately acquire spatial mapping information of real spaces. Spatial mapping is an operation that allows users to scan or capture spatial data and reconstruct it into mesh data, which can be used for further modification, spatial analysis, and visualization [7,8,9].

In construction, architecture, and urban design, practitioners are increasingly incorporating contemporary data visualization tools to depict their designs visually [1,10,11]. These technologies aid in data collection, current situation assessment, and the rapid, accurate creation of 3D content. Within the context of smart cities, enhancing human quality of life takes precedence [12,13,14]. Addressing human needs through problem-solving and human-centered design has become a shared objective among urban and architectural professionals. MR technology can serve as a versatile platform for various development applications, aiding designers in addressing the above context. Rapid mapping of existing interior spaces within a building enables users or clients to freely engage with their surroundings, envisioning their ideal home environment. This process serves as an effective initial survey step for projects, allowing architects to both capture 3D spatial data and grasp the client’s preferences. AR technology further facilitates the presentation of design proposals by consultants to clients. 

Despite the need for higher precision in professional projects, advanced scanning technologies such as terrestrial laser scanning (TLS), mobile laser scanning (MLS), and simultaneous localization and mapping (SLAM) are preferred. MR devices offer distinct advantages in certain applications to replace professional devices. Thanks to the advancements in computing, sensor technology, and 3D modeling tools, MR devices enable cost-effective exploration while allowing practitioners to achieve satisfactory results. When coupled with AR content, these devices prove invaluable for educational, research, and design endeavors [8,15,16,17]. Several MR devices can be used for these purposes, including head-mounted display (HMD) devices (HoloLens 2, Meta Quest 3, Apple Vision Pro, etc.) and mobile devices (iPhone Pro, ProMax 12 and later versions, iPad Pro 2020 and later versions). These devices are capable of capturing spatial information due to their built-in depth sensors [8,18].

Although MR technology has been researched and developed for a long time, its applications have not yet proven highly effective in professional fields requiring high precision, such as architecture, construction, and urban design [19,20,21]. However, the limitations of the spatial survey capabilities of commonly used MR devices have been noted, due to factors such as environmental changes, spatial configurations, material variations, lighting conditions, and operators’ skill. Additionally, the rapid pace of spatial exploration results in relatively low detail meshes generated by MR devices, thereby impacting the accuracy of spatial data [7,17]. A few studies have been conducted to test the accuracy of these devices across various contexts, aiming to identify the situations in which device performance meets the necessary standards. The research team noticed some gaps in previous studies. For example, some common factors in practical applications, such as the complexity of the survey environment, has been overlooked, and optimal level spatial mapping was only assessed through consideration of data variation [7,8,9,16,22].

Within this study, the research team selected HoloLens 2 and iPhone 13 Pro for the spatial mapping experiment. To enhance comparability, photogrammetry was introduced as an additional surveying and mesh construction approach. Photogrammetry is a popular method frequently used for collecting real-world spatial data. With advancements in supporting software and computing power, photogrammetry enables users to reconstruct highly accurate spatial data in an ideal environment [23,24,25]. There are two objectives of this research: **O1**—Evaluate the accuracy of spatial data collected by the solutions proposed in the article based on data variation; and **O2**—Calculate the performance of each method based on the following criteria: complexity of the environment, data file size, and data variation. From these evaluations, we discussed the suitability of each method for different application contexts.

To carry out this research and collect the data, three methodologies (spatial mapping with HoloLens 2, iPhone 13 Pro, and photogrammetry with a camera) were evaluated across three interior environments featuring distinct complexities. The outputs of these methods were compared against a ground truth model constructed using 3D modeling with precise dimensions, allowing for the assessment of each approach’s performance in different scenarios. The collected data were used to calculate performance for each method corresponding to the requirements set out in the scope of this study.

## 2. Materials and Methods

### 2.1. Spatial Mapping Operation with MR Devices

**Figure 1:**
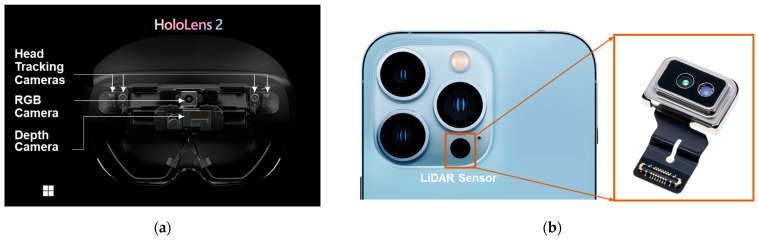
Spatial data collection with built-in depth sensor of MR device. (**a**) Depth camera inside HoloLens 2; (**b**) LiDAR sensor inside iPhone 13 Pro.

The HoloLens 2 is a state-of-the-art mixed reality (MR) headset developed by Microsoft, offering an immersive computing experience as an HMD device. Its enhanced optics provide a wider field of view and higher resolution, which significantly improve realism. Advanced hand tracking allows for intuitive interaction with holographic objects. Furthermore, HoloLens 2 is equipped with a range of sensors, including depth sensors, cameras, and inertial measurement units, which enable precise spatial mapping and tracking. This functionality allows 3D content to accurately anchor to real-world surfaces and environments, facilitating the seamless integration of digital content into the physical world. HoloLens 2 is utilized in various industries such as manufacturing, healthcare, architecture, design, and education, revolutionizing our visualization and interaction with digital content [9,18]. In this study, HoloLens 2 was employed for spatial mapping, enabling the collection and reconstruction of spatial data into a 3D model that can be exported for user analysis and utilization. The device’s primary function of collecting spatial data is facilitated by its long throw mode in conjunction with the built-in depth camera (Figure 1). By capturing and replicating the geometric features surrounding the device’s location, HoloLens 2 enables users to engage in interactive AR functionalities, including navigation, physics simulation, occlusion, and object placement [7,9,18]. Although Microsoft has announced the discontinuation of HoloLens 2 development, it remains a valuable HMD device for various research purposes. Launched and operated within the past five years, HoloLens 2 uniquely allows users to access and extract spatial data and then export it to other storage devices. This capability sets it apart from other popular HMD devices on the market, such as Meta Quest and Apple Vision Pro.

The iPhone features a light detection and ranging (LiDAR) sensor, a cutting-edge technology that significantly enhances depth perception and spatial awareness across various applications. The LiDAR sensor emits laser pulses to measure distances to objects, enabling more accurate AR experiences, faster autofocus under low-light conditions, and enhanced portrait photography with improved depth mapping. This advancement in sensor technology revolutionizes how users interact with their surroundings, providing new opportunities for immersive experiences and precise spatial mapping on mobile devices. Built-in LiDAR sensors are found in the following devices: iPhone Pro, ProMax 12 and later versions, and iPad Pro 2020 and later versions. For this research, the iPhone 13 Pro with a built-in LiDAR sensor was selected (Figure 1). However, an application from the iOS store must be installed to operate the spatial mapping experiment [8,26]. With the integrated technology on the iPhone, application developers continuously launch AR applications that support users in collecting and interacting with spatial data. This creates a seamless process, enabling users to collect, edit, and utilize realistic 3D objects within their environment.

### 2.2. Photogrammetry for Architectural Space

Owing to the advanced revolution in photogrammetry technology, including cameras and computer vision, rapidly reconstructing spatial data from images containing geometric data is now feasible, allowing even non-professionals to regenerate digital 3D models from real-life objects. The 3D digital content generated through photogrammetry can be easily modified, manipulated, and visualized directly inside 3D software (Unreal Engine, Blender, Unity, Sketchup, 3ds max, etc.), or used in rendered images, virtual environments, or various other types of 3D post-production. In the fields of architecture, construction, and urbanism, utilizing photogrammetry for surveying spatial data presents a cost-effective approach that yields dependable results suitable for professional projects, particularly when compared with other methods such as LiDAR sensors or laser scanning [23,24,25,27]. Given its proven effectiveness in collecting spatial data within these fields, photogrammetry emerges as a viable solution for comparison alongside the selected MR devices in this study.

### 2.3. Experimental Procedure

**Figure 2:**
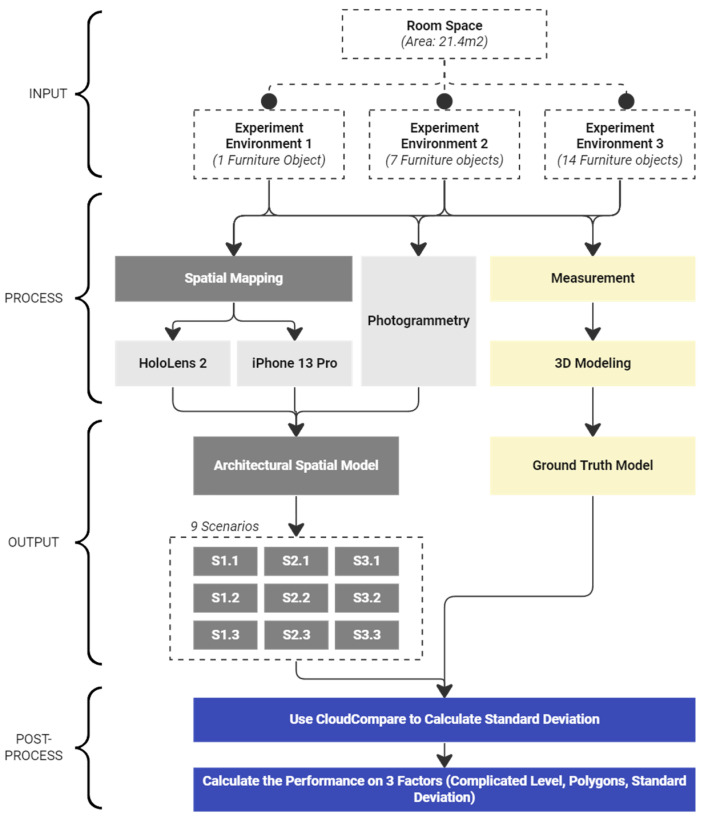
Experimental methodology and workflow.

The process of experimenting and evaluating the output results comprises four stages: input, process, output, and post-process (Figure 2). This sequence ranges from collecting input data in building environments using various solutions to the final assessment for comparison.

*Input stage:* In this phase, the research team selected a mutual space for the three different experimental environments. The overall structure and lighting conditions of the entire space remain consistent. Progressively, from environment 1 to 3, the complexity level escalates with the addition of more furniture within the room. Environment 1 contains one furniture object, while environments 2 and 3 house seven and fourteen different furniture objects, respectively.

*Process:* Firstly, all of the experimental environments and interior objects are measured carefully to construct 3D ground truth models with modeling software. Subsequently, each spatial survey method is operated to collect spatial data from every environment. HoloLens 2 and iPhone 13 Pro are used for real-time spatial mapping, while a mobile camera captures images of all details as input data for photogrammetry.

*Output:* In addition to the three ground truth models of the environments, nine different scenarios of spatial models are created using three methods across the three experimental environments.

*Post-Process:* The objective of this research is to optimize spatial mapping operations for the different contexts of architectural spaces. To achieve this, the nine different spatial models are compared with the ground truth model of their respective environment references. For instance, the ground truth model of environment 1 serves as a reference for the comparisons of spatial models 1.1, 2.1, and 3.1. Finally, using the comparison values, the research team assessed the performance based on three different factors: the complexity level of the environment, the total number of polygons in the spatial model, and the standard deviation between the spatial model and its reference.

### 2.4. Experimental Environment Preparation

**Figure 3:**
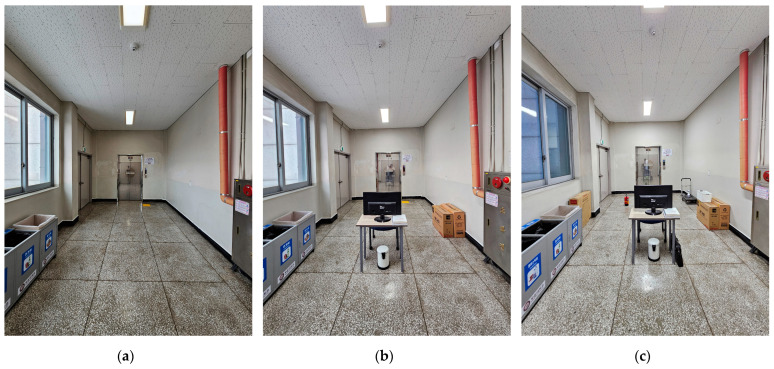
Experimental environment: (**a**) environment 1; (**b**) environment 2; (**c**) environment 3.

The selection of this particular space for experimental practice was based on its versatility for alterations and its manageable size, spanning approximately 20 m^2^. This size is ideal for practicing various spatial survey techniques such as spatial mapping and photogrammetry, striking a balance between being neither overly spacious nor exceedingly confined. Corresponding to a typical room size in common household designs, this space offers an ideal scale for experimentation. Furthermore, the room presents certain challenges for surveying due to the presence of specific materials such as metal, glass, and white-painted walls (Figure 3). These conditions serve as appropriate obstacles for devices utilizing depth sensors and for photogrammetry methods, testing their ability to navigate and map space effectively.

Regarding the experimental environment, the presence of windows could directly impact the quality of light in the room. Therefore, we chose to conduct the study on a cloudy day and utilized artificial lighting as the primary light source to ensure consistent lighting conditions. To enhance the intricacy of the experimental settings, a selection of furniture items was strategically positioned within the communal area of the room. Since the ground truth model was generated using traditional measurement methods by an architecture specialist, we selected spaces and items with uncomplicated shapes to minimize human error and ensure the ground truth model’s accuracy. The experimental space was kept simple, consisting of a room with consistent changes in furniture to ensure comparability of environmental complexity and avoid subjective comparisons. These items were arranged haphazardly, with a few items clustered together and others placed individually, thereby altering the overall dynamics of the environment. The chosen objects exhibited diversity in both size and shape, ranging from simple forms such as paper boxes to more distinct shapes such as plant pots and fire extinguishers. The list of items for each environment is detailed according to Table 1.

## 3. Results

### 3.1. Ground Truth Model with Measurement and 3D Modeling

**Figure 4:**
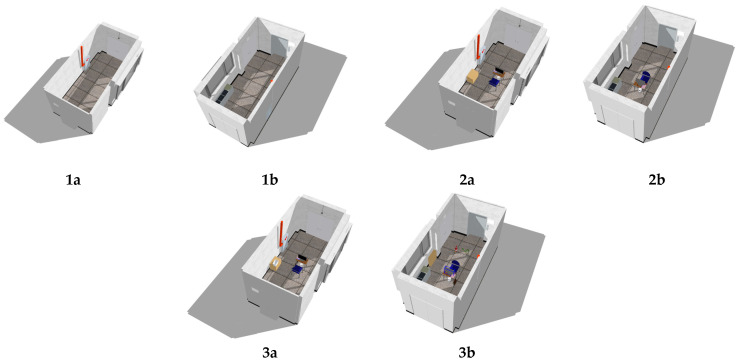
Ground truth models of three experimental environments: (**1**) experiment environment 1; (**2**) experiment environment 2; (**3**) experiment environment 3. (**a**) View from northeast; (**b**) view from southwest.

In alternative studies, ground truth models are constructed using specialized spatial data survey equipment such as LiDAR sensors or TLS devices. However, due to the constraints of this study, a more traditional method was employed. To construct the ground truth model, the research team used a conventional approach to survey the architectural landscape, involving traditional measurements and subsequent reconstruction of the space using 3D modeling software (Blender, Sketchup, 3ds max, etc.) (Figure 4). This endeavor demands meticulous precision and necessitates the involvement of seasoned professionals in the fields of architecture and interior design to participate in the measurement process.

This approach offers several advantages. Firstly, it effectively controls the polygon count in 3D models, ensuring that reconstructions of real spaces maintain a balance between polygon count and accuracy. Secondly, it addresses challenges posed by reflective surfaces such as glass or metal, ensuring accurate representation regardless of material properties. In this study, SketchUp software was used to create a ground truth model based on parameters measured from reality.

However, there are notable challenges associated with using this method to construct a ground truth model. Dimensioning lacks absolute precision, preventing an exact reproduction of spatial shapes. Additionally, minor local deviations from flatness, verticality, and other features are typically overlooked or generalized in dimensioning.

### 3.2. Spatial Mapping with HoloLens 2 and iPhone 13 Pro

Employing spatial mapping methods for spatial data collection, the research team utilized two devices, namely the HoloLens 2 and the iPhone 13 Pro, to facilitate MR experiences. Initially, during the exploration of the functionalities of HoloLens 2 at the start of the experiment, many applications in Microsoft’s library enabling spatial data collection had been discontinued. Consequently, the study resorted to using the Microsoft Portal to establish connectivity between the HoloLens 2 and the computer in developer mode. Upon activation, HoloLens 2 automatically initiates spatial mapping by scanning the surrounding environment and storing the acquired data in memory, thereby facilitating AR experiences for users.

**Figure 5:**
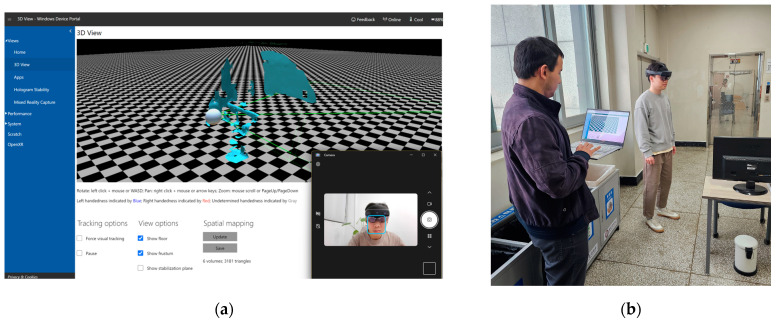
Operation of spatial mapping with HoloLens 2: (**a**) Microsoft portal interface; (**b**) room scanning process.

In the Microsoft Portal interface, users have convenient control over the spatial mapping operations of HoloLens 2, with the ability to comprehensively visualize the extent of collected spatial data. Opting to update information triggers changes in the spatial data based on the most recent scanning session of the HoloLens 2 device in the respective area (Figure 5). Users can iteratively update the data until they ascertain its currency and completeness. The implementation of HoloLens 2’s spatial mapping in this study is iterative and protracted, aimed at achieving optimal data collection. The spatial mapping resolution utilized by HoloLens 2 in this investigation was set at 500 triangles per cubic meter, which aligns with the device’s default mode. Subsequently, the spatial data obtained through the spatial mapping procedure can be immediately exported to a user’s computer as an OBJ file. This data can subsequently be readily utilized for tasks such as measurement and the creation of VR and AR environments, among others.

**Figure 6:**
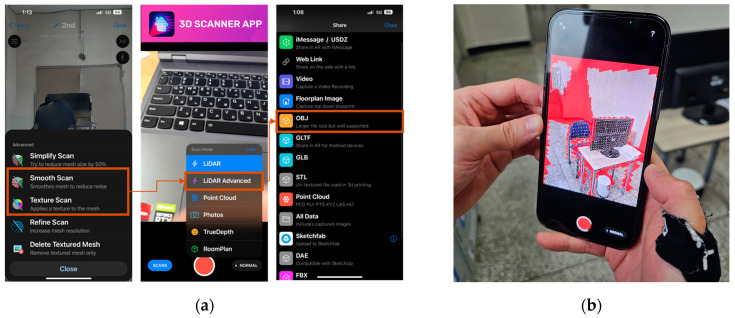
Operation of spatial mapping with iPhone 13 Pro and 3D scanner. (**a**) Settings for 3D Scanner. (**b**) LiDAR sensor is used to capture spatial information.

For spatial mapping tasks employing an iPhone 13 Pro equipped with a LiDAR sensor, the procedure for scanning the room to collect data is similar to that of the HoloLens 2. However, in this experiment, a third-party application called 3D Scanner (latest version) was utilized. This application allows users to acquire real-time spatial data akin to Near Eye Displays devices by leveraging the built-in LiDAR sensor. During the spatial survey process, users have the capability to continually monitor the collected spatial content and make appropriate adjustments. However, the immediate construction of 3D models is not feasible and requires a certain duration, contingent upon the desired content quality. In this investigation, the research team selected settings such as Smooth Scan, Texture Scan, and LiDAR Advanced. The final model is exported as an OBJ file (Figure 6). The experimenter scanned the entire room space using the content displayed on the phone screen, ensuring continuous updates until completion.

While the generation of a 3D model following real-time scanning is time-consuming, the 3D scanner software enables the iPhone 13 Pro to produce a model that incorporates the texture of the actual environment. Conversely, the mesh model produced by HoloLens 2 solely encompasses spatial data and lacks texture.

### 3.3. Photogrammetry and Mesh Construction

**Figure 7:**
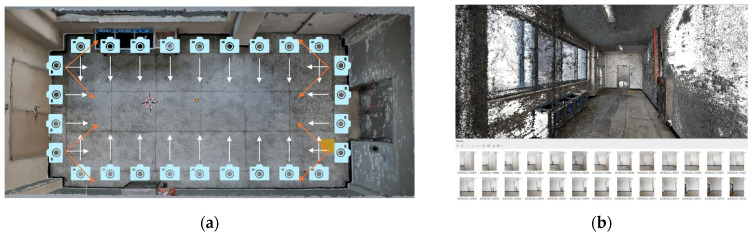
Operation of photogrammetry. (**a**) Sequence of images is acquired around the room space to capture spatial information as the point cloud mesh. (**b**) Point cloud mesh generated in Agisoft Metashape from a sequence of images.

Currently, photogrammetry is widely adopted as an economical method for spatial data acquisition. Utilizing the computational power of computers, photogrammetry techniques enable users to reconstruct 3D representations of diverse objects, ranging from small items to entire interior and exterior spaces, or even extensive areas. Numerous applications available on both mobile devices and computers have been developed to assist users in generating 3D models from sequences of images (Figure 7). In this study, images were captured using a mobile device (Samsung S23 ultra—ultra wide camera—12 MP) according to a specified protocol to ensure comprehensive data utilization before being imported into Agisoft Metashape software. Subsequently, the software analyzes the images to generate a point cloud mesh, which is further processed using algorithms to produce 3D models that can be exported as OBJ files.

**Figure 8:**
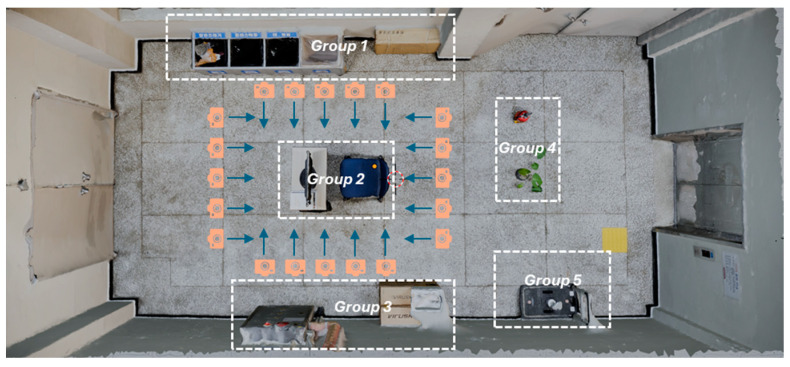
Sequences of images are acquired around each group of objects in the 2nd and 3rd experimental environments.

The quantity of photos captured varied, ranging from approximately 180 to 220 images, depending on the complexity of each research environment. Environments 2 and 3, which contained numerous objects in addition to open space, were categorized into distinct groups. Image sequences capturing the surroundings of these object groups were also acquired to ensure data processing accuracy using the Agisoft Metashape software (Figure 8). To prevent duplication of image data and facilitate point matching for seamless connectivity, photos were captured from specific distances, ensuring that various objects within the space were encompassed within each frame.

The 3D model generated through photogrammetry includes texture data, similar to the approach involving the iPhone 13 Pro. However, this method is comparatively more time-consuming than other techniques as it necessitates post-data collection processing using computer software (Agisoft Metashape). Moreover, due to the lack of real-time spatial mapping, users may experience difficulties in ascertaining the adequacy of the collected data during the process. Furthermore, another potential constraint involves the number of model faces created by this method.

The photogrammetry method enables the creation of high-resolution models, resulting in a very large number of faces. In this study, the research team maintained the original model values and did not decimate faces to ensure the highest possible accuracy of the spatial data. During the experimental process, we found that decimating faces not only reduced the clarity and quality of the model but also significantly compromised the accuracy of the collected data. In this study’s photogrammetry practice, control points were not employed to precisely determine the spatial data collection locations. Instead, various techniques in 3D modeling and spatial data processing were utilized to achieve the highest possible accuracy in comparisons. This approach may introduce some errors into the final results.

### 3.4. Generated 3D Models with Spatial Data of Different Methods

**Figure 9:**
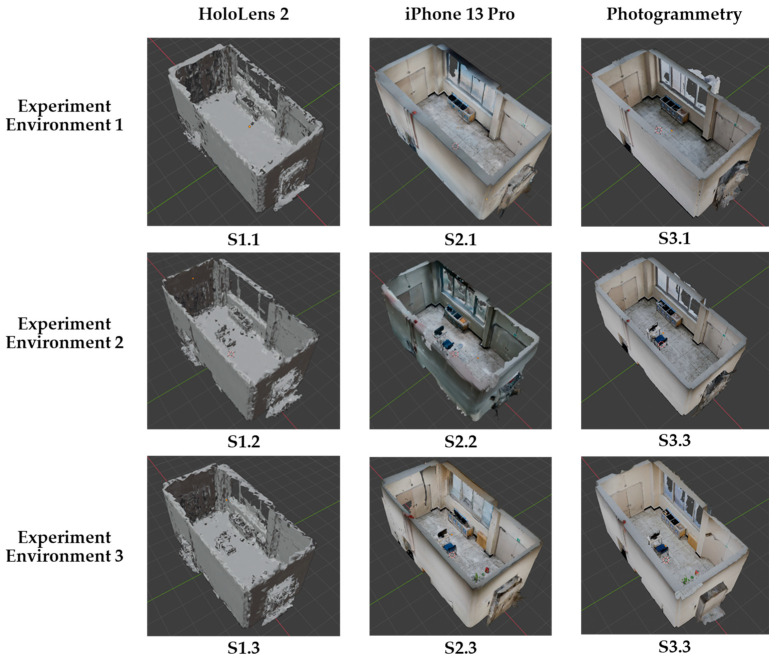
Generated 3D models with spatial data from different methods.

The results obtained reveal a clear distinction between the products created from three methods: spatial mapping using HoloLens 2, spatial mapping using iPhone 13 Pro, and photogrammetry (Figure 9). Certain differences are immediately recognizable through observation. Based on the output observations, iPhone 13 Pro was found to exhibit superior capability of collecting spatial information, particularly with reflective and metallic surfaces, compared with the other two methodologies.

Conversely, HoloLens 2 encounters challenges in identifying the spatial surfaces of glass windows and objects with reflective attributes such as metal. Furthermore, due to the default limitation of 500 triangles per cubic meter in HoloLens 2, the device struggles with recognizing objects of smaller sizes and those with intricate shapes (such as plants, fire extinguishers, and baskets), as evidenced in experimental environment 3. When comparing the outcomes of the photogrammetry and spatial model methods produced by the iPhone 13 Pro’s LiDAR sensor, substantial differences were not noted.

### 3.5. Using CloudCompare to Compare Generated 3D Models with Ground Truth Models

As outlined in the workflow and research methodology, in the post-processing phase of the experiments, to calculate the differences between 3D models created by three spatial mapping methods—using HoloLens 2, iPhone 13 Pro, and Photogrammetry—with ground truth models, the research team used CloudCompare software. This is an open-source software specifically used to edit, measure, and process spatial data.

**Figure 10:**
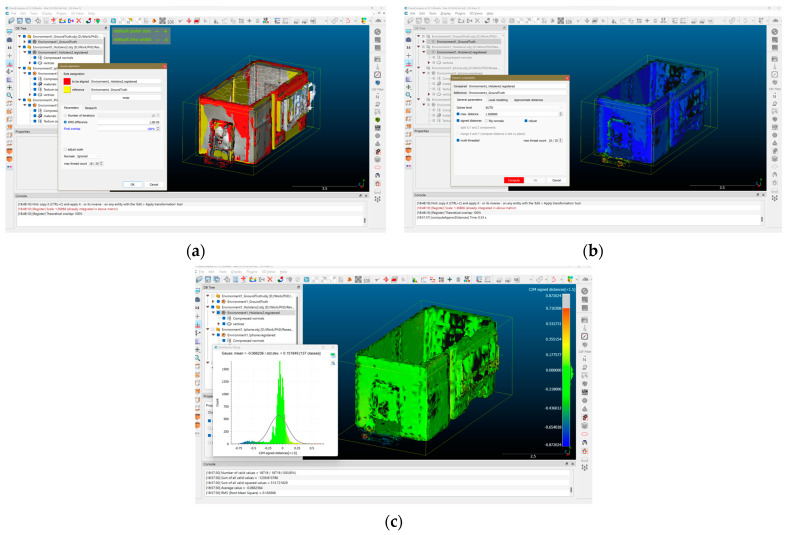
Interface of CloudCompare and the step-by-step calculation for model comparisons. (**a**) Cloud registration for aligning entities; (**b**) Distance computation for deviation; (**c**) Calculation of mean distance and standard deviation.

The two models to be compared were imported into CloudCompare as OBJ files. In all scenarios of comparing different environments in experiment environments 1, 2, and 3, the ground truth model was set as the reference model for rotation, scaling, movement, and distance variation computations. Firstly, the experiment practitioner adjusted the position and rotation of the model to be compared in order to match it with the ground truth model and subsequently proceeded with cloud registration. Through software calculations, the two models were perfectly aligned. The default parameters for the cloud registration process included 20 iterations, a root mean square difference of 1.0 × 10^−5^, 100% final overlap, and a random sampling limit of 50,000 (Figure 10a). Alignment can be adjusted along all three XYZ axes. All of the models in different experiment scenarios were treated individually.

Once the two models were aligned, the research team conducted a mesh distance computation, allowing the software to calculate two parameters: standard deviation and mean distance. This step involved comparing nine different scenarios, with the maximum distance selected for a comparison set at 1.5 m (Figure 10b). In the final step, after the variation of spatial data between the two models had been completely calculated, the research team supported the observations and assessment of the model’s spatial data accuracy comparison through two forms:

1. Spatial data variation heat map with parameters in the following order: Location difference at 1.5 m is red; position difference at 0.1 m is yellow; different positions at −0.1 m is green; different positions at −1.5 m is blue.
2. The diagram shows the variation level of the spatial data, with the x-axis being the distance difference parameter, and the y-axis is the number of faces/polygons of the model (Figure 10c). All model parameters in the nine scenarios of spatial data collection and 3D model reconstruction are shown in Table 2.

**Figure 11:**
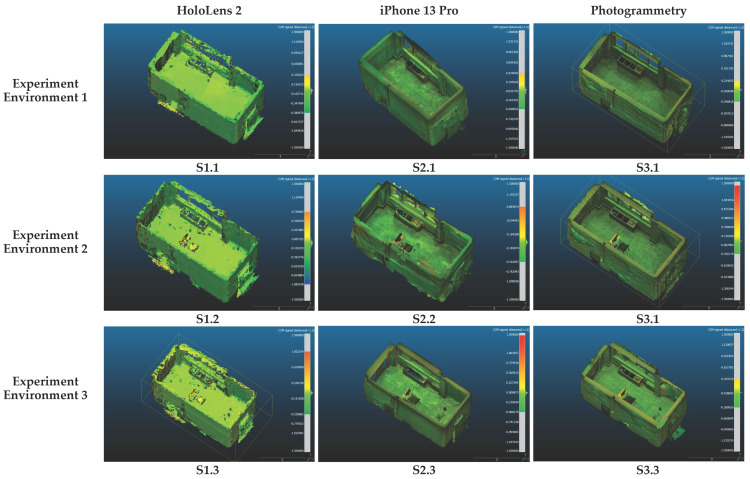
Spatial data variation calculation based on the comparison among created 3D models from different scenarios and ground truth models.

The final results of the comparison can be observed in Figure 11. All three methods performed efficiently in collecting spatial data in free-space environments, such as environment 1, which is less complicated. In the next two environments, all three methods demonstrated difficulty in reconstructing space in areas where interior objects are located. In general, across all spatial data collection instances, the collected spatial data tended to be a positive value. This indicates that when collecting spatial data via all three methods, inaccurately collected data typically scatters closer to the sensor or camera location rather than in the opposite direction. This finding contributes significantly to the navigation operation of AR content when using MR devices in this study, suggesting a need for improvements in proximity-based data handling for enhanced accuracy.

### 3.6. Standard Deviation Export via CloudCompare

This study primarily aimed to assess the optimization capacities of various devices in constructing environments suitable for AR interactions within the context of architectural design. Consequently, the generated 3D environments may not necessarily appear more realistic but are optimized for functional use in AR interactions. AR interactions require capturing precise spatial data for navigation and accurately rendering physical elements within real-space environments, as facilitated by MR devices. The collected environmental data must be adequate to create satisfactory conditions for effective AR interactions. While high-quality data collection may occasionally result in a significant increase in file volume, the enhancement in accuracy is not always proportionate.

The output models generated by the three different methods employed in this study exhibit varying numbers of faces/polygons for each research environment. Ranked in ascending order of faces/polygons, the methods are as follows: HoloLens 2, iPhone 13 Pro, and photogrammetry. The models produced by the iPhone 13 Pro contain approximately 2.5 times the number of faces/polygons compared with those generated by HoloLens 2. Conversely, models developed through the photogrammetry method feature 16 times the number of faces/polygons compared with the iPhone 13 Pro and approximately 40 times more than those from HoloLens 2 (Table 2). The results on the number of faces/polygons of the methods for all three experiment environments reveal that this number also increases as the complexity of the environment increases with an increase in the number of objects featuring special shapes.

As previously stated, assessing the adequacy of spatial data collected for constructing a 3D environment cannot solely rely on observation. Therefore, a comparison with the ground truth model is necessary to precisely evaluate spatial differences. In this study, mean distance (μ) (Equation (1)) and standard deviation (σ) (Equation (2)) calculations were employed to gauge the extent of variance between models generated by MR devices and real spatial data in meters (Table 2). These calculations were facilitated by CloudCompare software, which was utilized by the research team to compute these parameters based on the 3D models derived from all three experiments and the ground truth models. These analytical steps are critical in quantifying the effectiveness and precision of each method in various architectural settings.

(1) $$\mu =\frac{1}{N}\sum_{i=1}^{N}x_{i}$$

(2) $$\sigma =\sqrt{\frac{1}{N}\sum_{i=1}^{N}{\left(x_{i}-\mu \right)}^{2}}$$

*N*: Number of all distances; *x_i_* = Distance from point *i* to its reference.

Standard deviation is a statistical measure that quantifies the amount of variation or dispersion in a set of data points. It provides a numerical value indicating the extent to which individual data points differ from the mean (average) of the dataset. A higher standard deviation indicates greater variability among the data points, while a lower standard deviation suggests that the data points are closer to the mean. According to this interpretation, when utilizing standard deviation to assess the precision of spatial data collected in research methodologies, a lower value of this parameter corresponds to a higher level of data accuracy.

## 4. Discussion

The discussion based on the research tasks given at the beginning of this article is as follows.

**Figure 12:**
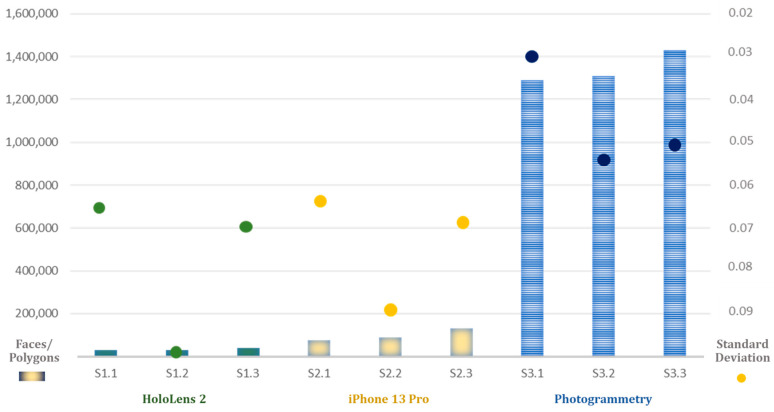
Spatial data mapping accuracy comparison among nine scenarios, with three data collection and model reconstruction methodologies. Note: faces/polygons defined as column; standard deviation defined as point.

**O1**—Evaluate the accuracy of spatial data collected by the solutions proposed in the article, based on data variation: The analysis indicates that across all three experimental settings (1, 2, and 3), the photogrammetry-generated model consistently exhibited the highest level of accuracy, as evidenced by its lowest standard deviation value. Conversely, the spatial data captured by the LiDAR sensor with the iPhone 13 Pro device slightly surpassed those obtained using the depth sensor of HoloLens 2. Notably, in experimental environment 1, the photogrammetry approach produced deviation data that was only half that of the other two methods. Interestingly, all three data collection methodologies yielded the highest standard deviation outcomes for environment 2, indicative of the least accuracy in this setting (Figure 12). As mentioned before, the number of faces/polygons of models reconstructed by the photogrammetry method was significantly higher than that with the other two methods. Consequently, the size of OBJ files created by this method was also substantially higher, which can present a challenge for this option in practical projects due to the increased demands of storage and processing capacity.

**O2**—Calculate the performance of each method, based on the following criteria: complexity of the environment, data file size, and data variation: To identify the most effective method for collecting spatial data to support the creation of AR interactive content in architectural design, several key factors must be considered. Merely ensuring the lowest standard deviation parameter in a scenario does not automatically qualify it as the optimal solution. In our study, we constructed three experimental environments of varying complexity, which serves as the first factor influencing the outcome of spatial data collection. We hypothesized that as the complexity of the environment increases, characterized by the presence of more intricately shaped objects, the number of faces/polygons in the ground truth model would likewise increase. Thus, the spatial data collection tools in our study performed most effectively in spaces predominantly comprising flat surfaces, while encountering greater challenges in the remaining two environments.

The second critical factor our research team considered in architectural space surveys was the accuracy of each method. This study assumed that models with lower standard deviation data demonstrated superior performance. Finally, storage resources represent a significant consideration, as they are determined by the number of faces/polygons comprising the reconstructed model. Constructing AR interactive content necessitates a balance between spatial information accuracy for navigation tasks and maintaining a compact data size. This ensures efficient resource utilization, prevents unnecessary data accumulation, and minimizes processing time on devices. The research team assumed that the productivity of the scenario is higher when the number of faces/polygons of the model is lower. According to this explanation, this study proposes a calculation equation for evaluating spatial mapping operation performance, referred to as Equation (3).

(3) $$P=\frac{F_{t}}{F\cdot \sigma }$$

*P*: Performance of the spatial mapping operation

*F_t_*: Total polygons of ground truth mode

*F*: Total polygons of comparing model/scenario

*σ*: Calculated standard deviation of scenario

Based on the standard deviation data collected through the CloudCompare application for each scenario and the number of faces/polygons of each model, the research team could effectively calculate the spatial mapping operation performance of different methods (Table 3).

The findings demonstrate that HoloLens 2 outperforms the other methods in spatial mapping across all three environments, achieving scores consistently threefold higher than those obtained using the iPhone 13 Pro’s built-in LiDAR sensor. While photogrammetry exhibits the most precise spatial data collection, indicated by its lowest standard deviation values across all experimental settings, its use within the study’s context is comparatively limited. This limitation primarily stems from the substantial number of faces/polygons it generates, which does not translate into a corresponding increase in spatial data accuracy.

HoloLens 2’s real-time spatial mapping exhibits commendable accuracy for immediate interaction with AR content. However, the 3D models derived from the spatial data gathered by HoloLens 2 lack versatility as they feature a constrained number of faces/polygons and lack texture data. Consequently, if architectural design endeavors necessitate usable 3D data for environment recreation, the iPhone 13 Pro proves to be a fitting and efficient device, balancing detail and real-time processing capability.

The photogrammetry approach, while effective in configuring environments for AR interactive content, involves a comparatively longer processing duration than the other two alternatives. The research team believes that this method can be efficiently employed in projects requiring meticulous reconstruction of real spaces into high-precision 3D models for architectural visualization, especially where the highest level of detail and accuracy is crucial.

## 5. Conclusions

In this investigation, a comparative analysis of spatial data collection and 3D model reconstruction was conducted across three distinct interior environments, each featuring varying degrees of complexity achieved through the arrangement of additional furniture groups. Three methodologies were employed: spatial mapping utilizing the HoloLens 2 depth sensor, spatial mapping leveraging the LiDAR sensor of the iPhone 13 Pro, and photogrammetry. This resulted in nine unique scenarios corresponding to nine 3D models reconstructed from the collected spatial data. The accuracies of these scenarios were evaluated by computing spatial data discrepancies using the standard deviation extracted from the CloudCompare application.

In assessing the optimal level of spatial mapping activities for the development of AR content in architectural design, the research team considered three pivotal factors directly influencing the outcomes. The first factor pertains to the complexity of the environment, gauged by the number of faces/polygons constituting the ground truth model. The second factor revolves around the precision of spatial data, evaluated through the standard deviation metric. Lastly, the capacity of the resulting 3D file is dictated by the number of faces/polygons comprising the model itself.

Following the computation of these parameters, the research team concluded that across all scenarios, encompassing both simple and intricate environmental setups necessitating spatial data collection, the HoloLens 2 device exhibited the most optimal performance within the parameters outlined in this study. Subsequently, the iPhone 13 Pro LiDAR sensor ranked next in efficacy, followed by the photogrammetry method. Different priorities may arise depending on the specific requirements of the intended 3D model. While the HoloLens 2 facilitates real-time spatial data collection with minimal storage demands, optimizing AR environment construction, the resulting spatial data lack texture data and feature a lower polygon count, rendering them unsuitable for post-processing architectural design operations. Conversely, users can generate a 3D model with marginally superior accuracy compared with HoloLens 2, supplemented with texture data, by utilizing the 3D Scanner application in conjunction with the built-in LiDAR sensor of the iPhone 13 Pro. Despite its advantages, this approach entails a significant increase in storage requirements, resulting in three times the number of faces and polygons compared with the HoloLens 2 device.

Conversely, the Photogrammetry method, while yielding the highest spatial data accuracy along with texture data inclusion, involves multiple processing steps across various software platforms, resulting in a 3D model with an excessively high number of faces and polygons. This method can be refined through various optimization techniques, particularly when the project objective involves accurately replicating the physical environment in virtual space. Users have the flexibility to employ various strategies during photogrammetry to eliminate redundant data, complemented by post-processing steps such as retopology and 3D modeling. However, compared to other options, photogrammetry requires more skill from the user, specialized software, and a long duration of practice.

This research still faces many challenges. Due to the lack of professional equipment necessary for high-accuracy surveying and spatial data collection, such as TLS, MLS, and SLAM, we employed traditional measurement methods by specialists to create a ground truth model. Consequently, the experimental environment was kept minimal with straight shapes to limit human error. Additionally, within the scope of this research article, the research team focused purely on comparisons conducted with developed comparison tools to determine the performance of devices based on various factors. The team was not focused on creating new software or algorithms for comparing spatial data.

This study paves the way for further exploration into the utilization of visualization technology in architectural design. Future research endeavors could focus on leveraging the 3D model outputs generated through the spatial mapping techniques outlined in this study to fulfill diverse visualization objectives such as: Improving design and operation support by integrating the project workflow; enhancing user interaction with the generated AR content; and optimizing the data handling processes. Studies on optimizing spatial data collection need to consider various factors within a real project workflow, not just the level of accuracy. Taking a holistic approach ensures that the chosen methods are practical and effective in real-world applications.

## Figures and Tables

**Table 1:** List of objects in three different experimental environments.

Components	Environment 1	Environment 2	Environment 3
Room	Total Area: 21.4 m^2^		
Furniture	**(1 Object)** Waste Storage	**(7 Objects)** Waste Container Boxes Table Chair Monitor Books Waste Bin	**(14 Objects)** Waste Container 2 Box Groups Table Chairs Monitor Books Waste Bin Plant Fire Extinguisher Basket Spray Bottle Hand Truck Bag

**Table 2:** Model information and deviation results for nine scenarios acquired from the CloudCompare calculation.

EE	Ground Truth Model		HoloLens 2 Model				iPhone 13 Pro Model				Photogrammetry Model			
	File Size (mb)	Faces	File Size (mb)	Faces	MD	SD	File Size (mb)	Faces	MD	SD	File Size (mb)	Faces	MD	SD
1	0.426	3161	2.19	30,655	−0.0356	0.0656	7.78	81,639	−0.0295	0.0634	195	1,289,965	0.0056	0.0301
2	1.77	10,035	2.23	31,468	−0.0328	0.1003	9.31	88,971	0.00009	0.0899	196	1,309,114	0.0180	0.0556
3	14.2	96,972	2.81	39,103	0.0063	0.0703	16.8	130,123	0.00933	0.0695	202	1,428,345	0.0188	0.0512

EE: experiment environment; SD: standard deviation (σ); MD: mean distance (μ), unit: meter.

**Table 3:** The performance calculation of all scenarios.

P	HoloLens 2 Model	iPhone 13 Pro Model	Photogrammetry Model
EE 1	**1.5711374**	0.610993	0.0814077
EE 2	**3.1802083**	1.2553794	0.1377818
EE 3	**35.287676**	10.718464	0.1371681

EE: experiment environment; P: performance calculation.

## Data Availability

The original contributions presented in the study are included in the article, further inquiries can be directed to the corresponding authors.
